# Reviewers in 2021

**DOI:** 10.1098/rsos.220230

**Published:** 2022-03-16

**Authors:** Wendy Hall

**Affiliations:** *Editor-in-Chief*

The last year was, much like its predecessor, filled with an extreme challenge for many around the world. Repeated waves of COVID infection and new variants continued to bring disruption and misery, though vaccines and vaccination strategies began to offer hope to societies of establishing a new normality.

It is in this context, then, that we offer thanks to our numerous reviewers for their time, dedication and expertise over the last year. The success of research journals like *Royal Society Open Science* depends on reviewers, and we remain very grateful to you all, especially considering the difficulties so many have faced over the last year (and more).

To provide an opportunity for recognition for reviewers, we list the names of all reviewers who have kindly contributed to the journal (unless they have opted out). This article is made permanent and citeable by its digital object identifier (DOI). We encourage you to quote your contribution in your applications for tenure, promotion and grants, or other forms of assessment. Where you have provided it, we have included your ORCID, so your contribution can be unambiguously assigned to you.

The team greatly appreciate all the work our reviewers do on behalf of the journal, and we look forward to working with you again in 2022.

Abarra I
Abbehausen C
Abbot D
Abd-AlGhafar W https://orcid.org/0000-0003-1489-4239
Abdel-Megied AM
Abe M
Abeler-Dörner L
Aboelhamd M https://orcid.org/0000-0002-9774-0587
Ackermans N https://orcid.org/0000-0001-8336-1888
Aczel B https://orcid.org/0000-0001-9364-4988
Adams JD
Adamson M https://orcid.org/0000-0002-8994-2170
Addleman D https://orcid.org/0000-0002-6667-322X
Adekunle A
Adler F
Agioutantis Z
Agliari E
Agren T https://orcid.org/0000-0002-1248-1310
Aguilar-Perera A
Aguilera V
Ahamed MS
Ahmed H
Ahmed J
Aiello LM https://orcid.org/0000-0002-0654-2527
Ak Khateeb S https://orcid.org/0000-0002-4250-0834
Akanda MR https://orcid.org/0000-0002-9529-0578
Akeroyd M
Akhmetzhanov A https://orcid.org/0000-0003-3269-7351
Akselrod M
Aladjem M
Alaux C
Albizu I
Aldred R
Alexandridis G
Alfani G
Alfonso I
Ali A
Alkan C
Allan A https://orcid.org/0000-0003-3495-8826
Allen B https://orcid.org/0000-0003-0282-6407
Almirantis Y
Alonso C
Alvares D https://orcid.org/0000-0002-6521-9148
Amato K
Amici F
Aminah NS https://orcid.org/0000-0002-2767-6006
Amos W https://orcid.org/0000-0002-0971-9914
Anco SC https://orcid.org/0000-0002-2125-3627
Anderson J
Andrada E https://orcid.org/0000-0002-9300-4056
Arabi A
Aranda E https://orcid.org/0000-0003-4550-343X
Aranda I
Aranda P
Araujo R https://orcid.org/0000-0002-3360-2214
Arellano C https://orcid.org/0000-0001-9672-3497
Arias P
Ariga K
Arzani A https://orcid.org/0000-0002-3706-7909
Aschi M
Ashraf Fahmy S
Attwa M
Authier M
Axfors C
Ayers J https://orcid.org/0000-0001-7722-0376
Ayoub B
Ayuso-Carrillo J https://orcid.org/0000-0002-9939-2125
Ayyappan A
Azarian T https://orcid.org/0000-0002-8611-5241
Badahe S
Baddeley A
Bagchi S
Baggio J
Baguley T
Bajardi P https://orcid.org/0000-0001-8865-4495
Bajda T
Bakker M
Balas B
Balcarcel A https://orcid.org/0000-0002-3826-834X
Baldini F
Ballesteros S
Balunaini U
Bansode U
Banzhaf W
Bao G
Baranger D
Barbosa JA
Bardella L https://orcid.org/0000-0002-6042-0600
Barek J
Bargal B
Bark R
Baron M https://orcid.org/0000-0001-9069-7638
Barrafrem K https://orcid.org/0000-0003-3171-8640
Barrett P
Barrios Garcia Moar MN
Barthelemy M https://orcid.org/0000-0003-0890-9713
Bastiaans D https://orcid.org/0000-0002-3096-2062
Bastidas D
Basu-Gupta S
Batsleer F https://orcid.org/0000-0001-5893-5966
Battista N
Bayro-Corrochano E
Bazzi M
Beatty B https://orcid.org/0000-0002-5464-0041
Beauchamp G https://orcid.org/0000-0002-3393-9562
Becerra M https://orcid.org/0000-0002-1123-7236
Bedos-Rezak BM https://orcid.org/0000-0001-6372-9008
Behera A
Beinart R https://orcid.org/0000-0003-1672-5957
Beitollahi H
Beja-Pereira A https://orcid.org/0000-0002-1607-7382
Belal F
Belal TS
Bell V
Benaroch P
Benhaïm D
Benítes A
Bennett T
Bentley B
Bento AI
Benton M https://orcid.org/0000-0002-4323-1824
Benvenuto C https://orcid.org/0000-0002-8378-8168
Berens S
Beresic-Perrins R
Berger-Tal O https://orcid.org/0000-0002-7396-456X
Bergman T https://orcid.org/0000-0002-9615-5001
Bermejo J
Bernardin AM
Berreau LM
Bestwick J https://orcid.org/0000-0002-1098-6286
Beu A
Beukeboom R https://orcid.org/0000-0002-7912-713X
Bezuidenhout H
Bhardwaj A
Bhattacharyya S https://orcid.org/0000-0003-3256-7688
Bhattarai A https://orcid.org/0000-0002-2648-4686
Bhowmik R
Bieler R
Bierbach D
Biondi LM
Bissas A
Biswas R https://orcid.org/0000-0001-9517-9485
Black A
Blackwell S
Blanco Bregón F https://orcid.org/0000-0003-1283-8313
Böckerman P https://orcid.org/0000-0002-5372-2985
Bode-Oke A https://orcid.org/0000-0002-2746-1822
Boehnke J
Bogovic J
Bolton K
Bomphrey R https://orcid.org/0000-0002-4748-0510
Bonetti L
Bonnet S https://orcid.org/0000-0002-5810-3657
Booth C
Bor D
Borah D https://orcid.org/0000-0002-9367-5589
Borcea C
Borg G
Bormashenko E https://orcid.org/0000-0003-1356-2486
Borrego N
Borstnar R
Bos K https://orcid.org/0000-0003-2937-3006
Botías C https://orcid.org/0000-0002-3891-9931
Botta F https://orcid.org/0000-0002-5681-4535
Bowman R
Bozhko YY
Bramstedt K
Brand T
Branney P
Branson R
Bray E https://orcid.org/0000-0002-3230-0636
Brazil A
Brehm L
Brestic M
Briefer E https://orcid.org/0000-0003-4147-0319
Briggs D https://orcid.org/0000-0003-0649-6417
Briz-Redón Á
Brlík V
Brocklehurst N https://orcid.org/0000-0003-2650-5480
Brogan A https://orcid.org/0000-0002-8361-6649
Brökel T
Bronzati M https://orcid.org/0000-0003-1542-3199
Brook C https://orcid.org/0000-0003-4276-073X
Brookes P https://orcid.org/0000-0002-8639-8413
Brooks J https://orcid.org/0000-0001-6700-8283
Brookshire G
Bruckner D
Brumm H https://orcid.org/0000-0002-6915-9357
Brundage C
Buades-Rotger M
Buck J https://orcid.org/0000-0003-3202-7665
Budd CJ
Bueno CG
Bugnyar T
Bullimore M https://orcid.org/0000-0002-6315-3720
Burant J https://orcid.org/0000-0002-0713-3100
Burch S https://orcid.org/0000-0001-8859-7603
Burton H
Butcher N
Butler R https://orcid.org/0000-0003-2136-7541
Butt N
Byrne H
Cai J
Cai l https://orcid.org/0000-0001-5976-2382
Calandra I
Callens SJP (Sebastien)
Cameron S
Campbell H https://orcid.org/0000-0002-0959-1594
Campos P https://orcid.org/0000-0003-1285-4671
Campos-Mercade P
Canamero L
Capraro V https://orcid.org/0000-0002-0579-0166
Cardillo A
Cardoso G https://orcid.org/0000-0001-6258-1881
Carignano A
Carr C
Carr T
Carré J https://orcid.org/0000-0002-8967-9288
Carroll E
Carvalho BM
Carvalho R https://orcid.org/0000-0002-2590-8686
Casalino G
Casero MC
Caspers B https://orcid.org/0000-0002-4380-0476
Castagnola V
Causin P
Cavallini Johansen I
Cavallini E
Cazzola D https://orcid.org/0000-0001-7877-6755
Ceci SJ
Çelik S
Celiku O
Cerna Bolfikova B http://orcid.org/0000-0001-8059-4889
Cerrato G
Cervantes E https://orcid.org/0000-0002-3874-1491
Cespi D
Chahl J
Chaine A
Chakrabarty S
Chakraborty S
Chamani J http://orcid.org/0000-0003-2562-2458
Chandrasekar S
Chan-Reynolds M
Chao DL
Charpentier C
Chartier C
Chatelain M
Chekanov K https://orcid.org/0000-0002-2699-6761
Chen D https://orcid.org/0000-0002-0471-8162
Chen H
Chen J-D
Chen X https://orcid.org/0000-0002-9129-2197
Chen Y
Chen Y https://orcid.org/0000-0003-2169-4673
Chen Z https://orcid.org/0000-0002-3500-9182
Cheng B https://orcid.org/0000-0002-6982-0811
Cheong KH https://orcid.org/0000-0002-4475-5451
Chevallier C
Chi H https://orcid.org/0000-0003-2985-730X
Chiastra C https://orcid.org/0000-0003-2070-6142
Chilvers B
Chin J https://orcid.org/0000-0002-6573-2670
Choi YH
Chowell G https://orcid.org/0000-0003-2194-2251
Christen JA
Chrnaki G
Chu X
Chumchal M
Ciccolella S
Ciesielski K
Ciliberto S
Cimini B https://orcid.org/0000-0001-9640-9318
Citron DT
Ciuonzo D
Clark C https://orcid.org/0000-0001-7943-9291
Clark T
Clarke R
Clavier N
Clegg R https://orcid.org/0000-0001-7241-6679
Clemente C https://orcid.org/0000-0001-8174-3890
Clifford S https://orcid.org/0000-0002-3774-3882
Clua E https://orcid.org/0000-0001-7629-2685
Coates M https://orcid.org/0000-0003-2843-1075
Coccia C
Coffin J
Cohen K
Colder Carras M https://orcid.org/0000-0003-0750-524X
Colegrove K
Colet P
Coll M-P
Colling L https://orcid.org/0000-0002-3572-7758
Collins C
Collins K https://orcid.org/0000-0002-3379-4201
Colom R
Colquhoun D https://orcid.org/0000-0002-4263-017X
Colston T https://orcid.org/0000-0002-1127-1207
Comstock D
Conlan A https://orcid.org/0000-0002-2593-6353
Convertino M https://orcid.org/0000-0001-7003-7587
Conway P
Cook A
Coombs D https://orcid.org/0000-0002-8038-6278
Cooper K
Cooper M
Corker KS https://orcid.org/0000-0002-7971-1678
Cornelissen P
Coscia M https://orcid.org/0000-0001-5984-5137
Costantini F
Cottineau C
Coutu A
Cox S
Crame J
Crespi B https://orcid.org/0000-0002-0362-392X
Cristofolini L https://orcid.org/0000-0002-7473-6868
Cropper S
Cross P https://orcid.org/0000-0001-8045-5213
Crowdy D https://orcid.org/0000-0002-7162-0181
Cucuringu M
Cuesta Ciscar A https://orcid.org/0000-0003-4243-1848
Cui H http://orcid.org/0000-0003-1686-5239
Cullen T https://orcid.org/0000-0002-4261-1323
Culling J
Cummings M https://orcid.org/0000-0002-4873-5378
Czaczkes T https://orcid.org/0000-0002-1350-4975
Czeszumski A
D'Août K
Dahm M
Daley A
Dallaston M
Dalmaschio C https://orcid.org/0000-0002-3773-5786
Dalmaschio CJ
Danel S https://orcid.org/0000-0003-0233-2343
Darroudi M
Das A
Das T https://orcid.org/0000-0002-6576-5552
Daversin-Catty C
Dawes J
Dawid P
Day A https://orcid.org/0000-0001-5819-9604
De Assis T
De Barros LD
De Castro A
De Gennaro L https://orcid.org/0000-0003-3613-6631
De Gregorio C
De Hevia M-D
Dean M
Dececchi A
Dediu D https://orcid.org/0000-0002-0704-6365
Deffner D https://orcid.org/0000-0002-1649-3861
DeFries R https://orcid.org/0000-0002-3332-4621
Degutis J
Dehmamy N
DeJong M
Del Campo J
Deng M
Denham AJ
Deokate RJ
Descamps S https://orcid.org/0000-0003-0590-9013
Di Bartolomeo A
Di Sarli V
Di Stefano A
Diaconescu AO
Dias L
Diego X
Dienes Z https://orcid.org/0000-0001-7454-3161
Difallah D
Dijkerman C https://orcid.org/0000-0002-5364-3312
Ding F
Dixon D
Dixon J
Dixson B https://orcid.org/0000-0003-0911-1244
Djaafara B
Djordjevic M
Dobson A
Doerig A
Dorfmann L https://orcid.org/0000-0002-9665-0272
Dorgan K https://orcid.org/0000-0002-9687-9675
Dörrenberg S https://orcid.org/0000-0003-3489-5001
Doublet V
Douglas R
Doupe D
Douroumis D https://orcid.org/0000-0002-3782-0091
Dowling D https://orcid.org/0000-0003-2209-3458
Dreber A https://orcid.org/0000-0003-3989-9941
Drenkova-Tuhtan A https://orcid.org/0000-0002-5163-7322
Drichoutis A
Drieu L https://orcid.org/0000-0002-7324-4925
Drohojowska J
Drysdale T
D'Souza A
Du J
Du J https://orcid.org/0000-0002-4309-9528
Duan H
Duan X
Dudschig C https://orcid.org/0000-0002-7041-7798
Duffy D
Dunmore C
Dunn R https://orcid.org/0000-0003-0927-2734
Dunning D
Dye C https://orcid.org/0000-0002-2957-1793
Dys SP https://orcid.org/0000-0001-8103-7603
Eben C https://orcid.org/0000-0001-9423-1261
Eckert K
Edmonds B
Ehrmann A
Eimer M
Ekdale E
El Ibrahimi B
Elbaum R https://orcid.org/0000-0003-4417-3811
Eley T
El-Gendy N
Elias P
El-Khateeb MA
Elqayam S
El-Shishtawy R
Elwakeel KZ
Emami Tabrizi I https://orcid.org/0000-0002-9825-4476
Endler J https://orcid.org/0000-0002-7557-7627
Engelhardt B https://orcid.org/0000-0002-8370-1317
Erhard F
Esquivel-Muelbert A
Esterhuyse S https://orcid.org/0000-0001-7675-443X
Esteves da Silva JCG
Estrada-Rodriguez G
Ezcurra M https://orcid.org/0000-0002-6000-6450
Fabregat C
Fairchild B
Fan H
Fang Y
Farrar BG
Farris D https://orcid.org/0000-0002-6720-1961
Farrugia N
Faure R
Feng Y
Ferguson HJ
Ferrante E https://orcid.org/0000-0002-2213-8356
Ferrante M
Ferrer M
Ferrie G https://orcid.org/0000-0003-1283-1402
Festa G
Fidino M
Fiehler K
Field S https://orcid.org/0000-0001-7874-1261
Filipiak M https://orcid.org/0000-0001-7438-4885
Fillon A
Finkel O
Finn A
Fischer J https://orcid.org/0000-0002-5807-0074
Fisher H https://orcid.org/0000-0001-5622-4335
Fleming S https://orcid.org/0000-0003-0233-4891
Fleming C
Foad C https://orcid.org/0000-0003-0423-2848
Foffa D
Forbes L https://orcid.org/0000-0002-9135-3594
Forde J
Fornberg B https://orcid.org/0000-0003-0014-6985
Forstmeier W https://orcid.org/0000-0002-5984-8925
Fossion R https://orcid.org/0000-0001-8456-2075
Fosso O
Fouda M
Fox M
Fox S
Francis AL
Francis G http://orcid.org/0000-0002-8634-794X
Frank-Ito D
Franklin E https://orcid.org/0000-0003-2754-596X
Frasier K
Freakley S
Fredensborg B
Freeman E
Fritze M https://orcid.org/0000-0002-6999-2840
Fröber K
Fröbisch J
Frömer R https://orcid.org/0000-0002-9468-4014
Frouin-Mouy H
Fu D https://orcid.org/0000-0001-9488-6989
Fu H-Y
Fuchs X
Fuess L https://orcid.org/0000-0003-0197-7326
Fuhse J
Fujii J https://orcid.org/0000-0003-4794-479X
Fujino T
Funston G https://orcid.org/0000-0003-3430-4398
Fyfe A https://orcid.org/0000-0002-6794-4140
Gagare S
Gagliardo A https://orcid.org/0000-0003-0214-3817
Gaillard J-M
Galán J https://orcid.org/0000-0003-3360-7602
Galbraith JW
Gale A
Gallou F
Gandy M
Gangoso L
Gao D
Gao H https://orcid.org/0000-0001-6852-9435
Gao J https://orcid.org/0000-0001-6659-5770
Gao M-Z
Garba S
Garcia G https://orcid.org/0000-0003-2995-9226
Garcia-Leon M
Gardas RL
Gardner J
Garnham L https://orcid.org/0000-0003-2185-1384
Garnier S https://orcid.org/0000-0002-3886-3974
Gathergood N
Gauthier C
Gavrilets S https://orcid.org/0000-0003-1581-4018
Gee B https://orcid.org/0000-0003-4517-3290
Geiker M
Gelens L
Gelman A
Germain D https://orcid.org/0000-0002-7608-2212
Gerothanassis I
Ghaffary A
Ghaghada K
Ghosh A
Giraldo J
Girard-Buttoz C https://orcid.org/0000-0003-1742-4400
Giribet G https://orcid.org/0000-0002-5467-8429
Giudici P
Gjesfjeld E https://orcid.org/0000-0003-1043-9810
Goehring L https://orcid.org/0000-0002-3858-7295
Goel H
Goenka A
Goncalves A https://orcid.org/0000-0001-7062-1900
Gong J
Gong Q
González-López V
Gopalkrishnan M
Gopolan N
Gordon R
Gostic K https://orcid.org/0000-0002-9369-6371
Gostoli U https://orcid.org/0000-0002-2394-3613
Gowtham B https://orcid.org/0000-0002-8817-072X
Gracia-Lázaro C https://orcid.org/0000-0002-9769-8796
Grainger S https://orcid.org/0000-0003-1475-835X
Grandal D'Anglade A
Grandini CR https://orcid.org/0000-0002-3336-309X
Granqvist P
Grant W
Graso M
Grassi L
Grassi M
Gratton C
Gravel-Miguel C https://orcid.org/0000-0003-1324-7937
Grebogi C
Green A
Green KC https://orcid.org/0000-0001-9122-885X
Greggor A https://orcid.org/0000-0003-0998-618X
Grenning A
Griffin C
Griffin S
Griffith D
Griffith S
Griffiths N https://orcid.org/0000-0002-6406-8632
Grima JN https://orcid.org/0000-0001-5108-6551
Grimes V
Grove M
Groves R https://orcid.org/0000-0001-9169-9256
Grozinger C https://orcid.org/0000-0001-5738-1201
Grüschow M
Guadagnini M
Guani Quinones Lebron S
Guenther A https://orcid.org/0000-0002-2350-0185
Guerrero Gonzalez A https://orcid.org/0000-0002-1556-9860
Guest O https://orcid.org/0000-0002-1891-0972
Guidolin L
Guimarães MS
Guinet C
Gulbiński W https://orcid.org/0000-0001-9771-7451
Guo B
Guo H
Guo Y https://orcid.org/0000-0002-4985-7553
Guo Z
Gupta S
Gupta V
Gurarie D https://orcid.org/0000-0002-5314-7888
Gurka R https://orcid.org/0000-0002-8907-6663
Gustison M
Gyorgy E
Hachmann W
Hacioglu S
Hadjikakou S
Haegeman B
Hagen S
Haifig I
Haigh M
Hakkenberg C
Halawa M https://orcid.org/0000-0001-6358-2691
Hall M https://orcid.org/0000-0003-4273-980X
Halliday D https://orcid.org/0000-0001-9957-0983
Hamada A
Han D
Han F https://orcid.org/0000-0003-3399-4008
Han H https://orcid.org/0000-0003-4668-3065
Han Y-H
Han Z
Hanel P
Harari AR
Hardacre C
Hardwicke T https://orcid.org/0000-0001-9485-4952
Harko T
Harper D
Harrison P
Hart L
Harvey C https://orcid.org/0000-0002-2830-0844
Harvey V
Harwood L
Hauber M https://orcid.org/0000-0003-2014-4928
Hauck K
Haug C
Hauser O
Hayashi Tang M
Hayman D https://orcid.org/0000-0003-0087-3015
Haynie HJ
Hayyiratul F
Haznar-Garbacz D
He B
He G
He X
Hecht E
Hedger SV
Heesen R https://orcid.org/0000-0003-3823-944X
Heirene R
Hellweger F
Helm C https://orcid.org/0000-0002-0308-8402
Hemery M
Hemraj AD
Henderson M https://orcid.org/0000-0002-2861-8668
Hengel E
Herrera J https://orcid.org/0000-0002-0633-0575
Hersh T
Heunis S
Heyd D
Heydecker B
Heynderickx P
Higham D https://orcid.org/0000-0002-6635-3461
Hilbe C
Hillen T
Hills T
Hilton C https://orcid.org/0000-0001-9113-1222
Hinch J
Hitam MS
Hitzer E
Hoadley K
Hobbs C https://orcid.org/0000-0002-2324-5882
Hodgkinson J
Höfling T
Hohol M https://orcid.org/0000-0003-0422-5488
Hojsgaard D https://orcid.org/0000-0002-8709-4091
Holcombe A https://orcid.org/0000-0003-2869-0085
Holl K
Holme P https://orcid.org/0000-0003-2156-1096
Holser R https://orcid.org/0000-0002-8668-3839
Homs Dones M https://orcid.org/0000-0002-8528-8140
Honda H
Hong L
Hooghiemstra H
Hormigo J
Horowitz J https://orcid.org/0000-0002-2643-5200
Horsley S
Horvath G
Hossain A
Houdijk H
Houlbreque F
Hovestadt T https://orcid.org/0000-0001-7368-6013
Howell T
Hu K
Hu Z
Hu Z
Huang Q https://orcid.org/0000-0001-6994-1739
Huang S
Huang Y
Huang Z
Huang Z-G https://orcid.org/0000-0001-9648-3067
Hubbard J https://orcid.org/0000-0002-0405-9398
Hughes A https://orcid.org/0000-0003-2677-1965
Hui L
Hülür G
Hundt PJ
Hurford A https://orcid.org/0000-0001-6461-5686
Hussain A
Hussain S
Hussein R https://orcid.org/0000-0001-9523-9388
Hutchins K https://orcid.org/0000-0001-8792-2830
Huveneers C
Hyodo F
Iacoviello D
Ibrahim A
Ichihashi N
Iglesias S
Ikram S
Imhoff R
Ingenloff K
Iqbal A
Isager P
Isik T
Islam Z-U
Ito T
Ito Y
Itzchakov G https://orcid.org/0000-0003-1516-6719
Ivanovska A https://orcid.org/0000-0001-6846-9583
Iwanicki T
Jack R
Jack R https://orcid.org/0000-0003-0086-4573
Jackson B
Jacob CR
Jaeger G
Jahan F https://orcid.org/0000-0002-0300-6315
Jain H https://orcid.org/0000-0002-9662-0444
James A https://orcid.org/0000-0002-1543-7139
James S
Jana B
Jani A https://orcid.org/0000-0001-8719-2962
Janiga G
Janis C https://orcid.org/0000-0002-2308-0520
Javadi A-H https://orcid.org/0000-0003-0569-6441
Javarone MA https://orcid.org/0000-0003-0514-9544
Jay C
Jeglinski J https://orcid.org/0000-0002-7997-6756
Jejurikar S
Jenner A
Jennings B
Jennings S https://orcid.org/0000-0003-2995-8813
Jensen K https://orcid.org/0000-0001-8644-1680
Jiang L
Jiang N
Jiang Z
Jijon-Alban S
Jimenez J
Jiménez S
Jin L
Jin Z
Johnson L
Johnson M
Johnston D
Johnston S https://orcid.org/0000-0003-4532-5573
Jolij J
Jones B https://orcid.org/0000-0001-7777-0220
Josens R
Joshi N
Jukar A
Jurenka R https://orcid.org/0000-0002-5090-3203
Kabała C
Kaczka D
Kadrekar R
Kaklis K
Kalan A
Kaltenberg A
Kamaruzzaman NF
Kamhi JF
Kan K
Kanakogi Y
Kaneti YV https://orcid.org/0000-0003-2433-7305
Kapadia M
Kappeler P https://orcid.org/0000-0002-4801-487X
Kara HE
Karahan HE
Karakashev S
Kashyap H
Katzner T https://orcid.org/0000-0003-4503-8435
Kaye L
Kear B https://orcid.org/0000-0002-3128-3141
Keçebas¸ A
Keles C
Kelleher F
Keller M https://orcid.org/0000-0001-9668-6817
Kelley S
Kelly C https://orcid.org/0000-0001-8253-357X
Kelly F
Kendall L https://orcid.org/0000-0002-0671-0121
Kenessov B
Kenett D
Kenkel C
Kenna R https://orcid.org/0000-0001-9990-4277
Kenny A
Kenward B
Keplinger T
Kerr C
Kettenbach A
Khan MdAS
Khan S https://orcid.org/0000-0003-4557-238X
Khan U
Khandare L
Khanehbad M
Khare R
Khatib F
Kheawhom S https://orcid.org/0000-0002-3129-2750
Kibblewhite M
Kibler A
Kicheva A
Kiefer M
Kier W
Kim H
King H https://orcid.org/0000-0003-3299-5690
Kinoshita S
Kirk P
Kirkley A
Kirkman S https://orcid.org/0000-0001-5428-7375
Kitchener A https://orcid.org/0000-0003-2594-0827
Kitture R
Kivell T
Klein R https://orcid.org/0000-0002-8713-1972
Klein S
Kleshch V
Kmita A
Knaub J
Knottenbelt W https://orcid.org/0000-0002-8490-1011
Koehler G
Koehn A
Koens L
Koepfli K-P
Koeshidayatullah A
Kohri M https://orcid.org/0000-0003-1118-5568
Kolář F https://orcid.org/0000-0002-8793-7992
Kolarik A https://orcid.org/0000-0002-5121-7512
Kolekar S
Konietzko-Meier D
König B
Korb S
Kordestani M
Korn C
Koski S
Koua FHM https://orcid.org/0000-0001-8371-9587
Kovacs K
Kovarik J https://orcid.org/0000-0002-0436-929X
Kowshik M
Krasheninnikova A https://orcid.org/0000-0001-8566-8277
Krebs N
Krieger-Redwood K https://orcid.org/0000-0003-4143-1168
Krishnan K
Krishnan S https://orcid.org/0000-0002-6466-141X
Krupp D
Kubáň P
Kucan K https://orcid.org/0000-0002-5123-0733
Kucharski A
Kuhlen A
Kujala J
Kumar Barui A
Kumar A
Kumar G
Kumar SC
Kumar S
Kumara S
Kundalwal SI
Kuperman M
Kuper-Smith B https://orcid.org/0000-0002-2565-222X
Küpper F
Kupper C
Kuyken W
Kvarnemo L
Kwapich C https://orcid.org/0000-0001-5141-7605
Kylliäinen A
Ladher R
Lago CL
Lagona F https://orcid.org/0000-0003-3364-3400
Laird AR
Lakens D https://orcid.org/0000-0002-0247-239X
Lakin M https://orcid.org/0000-0002-8516-4789
Lambert O https://orcid.org/0000-0003-0740-5791
Lambiotte R https://orcid.org/0000-0002-0583-4595
Landi S
Landry M
Lane P
Lane S https://orcid.org/0000-0002-3797-3178
Lange W-G
Lannig G
Larson G https://orcid.org/0000-0002-4092-0392
Larsson M https://orcid.org/0000-0003-4164-6513
Lattanzi A https://orcid.org/0000-0001-7540-3136
Lauprasert K
Le Meillour L
Le TB
Lea S https://orcid.org/0000-0002-8498-4187
Leclerc C
Lee A
Lee A
Lee E
Lee E
Lee M
LeFebvre M https://orcid.org/0000-0002-1741-9997
Legendre L https://orcid.org/0000-0003-1343-8725
Lekic B
Lemaitre J https://orcid.org/0000-0002-2677-6574
Leng T
Lenormand M https://orcid.org/0000-0001-6362-3473
Lepczyk C https://orcid.org/0000-0002-5316-3159
Leslie. D
Lester C
Lester E http://orcid.org/0000-0003-2207-5225
Leung J
Levine T
Lewandowsky S
Lewis L
Lewis P
Li A
Li B
Li B https://orcid.org/0000-0002-3792-2469
Li C
Li C https://orcid.org/0000-0001-7516-3646
Li C
Li D https://orcid.org/0000-0001-8269-7734
Li J
Li J https://orcid.org/0000-0002-8220-049X
Li K https://orcid.org/0000-0002-6699-6264
Li W
Li W
Li X https://orcid.org/0000-0001-6119-7757
Li Y
Li Y
Li Y
Li Z https://orcid.org/0000-0003-0103-2760
Libertus K
Lin B
Lin H
Ling H https://orcid.org/0000-0001-9014-7126
Lingle S
Lisicki M
Liu B
Liu B
Liu G
Liu J
Liu K
Liu L https://orcid.org/0000-0002-5760-2964
Liu X https://orcid.org/0000-0002-4422-4108
Liu Y
Liu Z
Loconsole M
Loewen M
Lomax B
London AJ
Lonsdorf E https://orcid.org/0000-0001-8057-401X
López-Arbarello A https://orcid.org/0000-0002-2924-3319
Lorenz C
Lorenzo IT https://orcid.org/0000-0002-5582-5523
Losic D
Lou Y http://orcid.org/0000-0003-3864-2001
Lourenco S
Lovas-Kiss A
Loveday C
Lowenstern J https://orcid.org/0000-0003-0464-7779
Lu K https://orcid.org/0000-0003-1370-8633
Luan H https://orcid.org/0000-0003-0722-1108
Luberti F
Luo F
Luo G
Lüpold S https://orcid.org/0000-0002-5069-1992
Lutz A
Lynam JM
Lyons N https://orcid.org/0000-0001-8187-986X
Lyons T
Ma M
Maclean I https://orcid.org/0000-0001-8030-9136
Macnair W https://orcid.org/0000-0001-6357-0834
MacNulty D
MacPherson SE
Maggi F
Maguire E
Mahmoud S
Maibaum T https://orcid.org/0000-0002-6627-5889
Majdandžić J
Malik MI https://orcid.org/0000-0001-6942-0407
Malkowski P https://orcid.org/0000-0003-0870-438X
Mamaev I
Mamlok-Naaman R
Mammino L
Mancini Teixeira H
Manonukul A
Manova D
Marcelino R https://orcid.org/0000-0001-8717-3243
March DS
Marcinkowska A
Mari L https://orcid.org/0000-0003-1326-9992
Marini M
Mário G
Mark C
Markuszewski MJ
Marquez E https://orcid.org/0000-0002-7503-1528
Marsh A
Martin A https://orcid.org/0000-0002-2843-6928
Martin B
Martin N
Martin-Duran J https://orcid.org/0000-0002-2572-1061
Martinez J-N
Mastrostefano E
Mathger L http://orcid.org/0000-0002-0603-0345
Mathur M
Mattsson C
Maussang F
May RF
Mazerolle L
Mazivila SJ
Mazurek A
Mazza V https://orcid.org/0000-0002-1634-3417
McAuliffe K https://orcid.org/0000-0002-4230-0250
McClannahan K
McClintock J
McClintock P
McCormick C https://orcid.org/0000-0001-7326-2560
McCulloch GA. https://orcid.org/0000-0003-1462-7106
McDermott S
McIntyre T https://orcid.org/0000-0001-8395-7550
McKinley G
Meares T
Meghwal M
Mehl S
Melin A https://orcid.org/0000-0002-0612-2514
Mellor D https://orcid.org/0000-0002-3125-5888
Meloni S https://orcid.org/0000-0001-6202-3302
Men Z
Mende-Siedlecki P
Menezes R
Meng Y
Mensinger A
Merey S
Mesoudi A https://orcid.org/0000-0002-7740-1625
Miguel M
Milano F
Milavec M
Miles E
Militao T https://orcid.org/0000-0002-2862-1592
Miller A https://orcid.org/0000-0002-3099-1648
Miller R
Millman R
Miolo G
Miras H
Mishuris G https://orcid.org/0000-0003-2565-1961
Mistriotis A
Mitchell K https://orcid.org/0000-0002-3921-0262
Mittal R
Miyakawa H https://orcid.org/0000-0002-3361-8443
Miyazaki T
Mizera A
Modrák M https://orcid.org/0000-0002-8886-7797
Mohan M
Mohtar R
Molho C https://orcid.org/0000-0002-2846-2544
Momin MM
Mönch C https://orcid.org/0000-0002-6531-6482
Monga B
Mongiardino Koch N https://orcid.org/0000-0001-6317-5869
Montefeltro F
Montefinese M https://orcid.org/0000-0002-7685-1034
Moore S
Morningstar M
Morris CD
Mostaço-Guidolin L https://orcid.org/0000-0001-6823-2965
Motallebzadeh H
Mueller M
Mukherjee M https://orcid.org/0000-0002-6421-4900
Mula O
Müller B
Muller J-M
Mumby H
Murall CL https://orcid.org/0000-0002-1543-4501
Murdock D https://orcid.org/0000-0001-6838-5566
Murgatroyd M
Murray B https://orcid.org/0000-0002-8527-3504
Müser M
Myalska H
Mysterud A https://orcid.org/0000-0001-8993-7382
Nabavi E
Nagai K https://orcid.org/0000-0002-8286-3111
Nakagawa Y
Nande A https://orcid.org/0000-0003-1726-6633
Narayanan H
Navarro-Noya YE
Nehlich O
Nelson C
Newman C https://orcid.org/0000-0003-0795-5435
Newsome T
Newton G
Nganvongpanit K
Ngonghala C https://orcid.org/0000-0002-8441-9495
Nguyen N
Nguyen T-V https://orcid.org/0000-0003-0777-4204
Nhamo L
Ni G https://orcid.org/0000-0002-9240-3020
Nicol C https://orcid.org/0000-0001-6734-2177
Niedenthal P
Nieto JJ
Nijhof A https://orcid.org/0000-0002-4153-1284
Nikolaidis T
Nikolic A https://orcid.org/0000-0003-1907-1565
Nilsonne G https://orcid.org/0000-0001-5273-0150
Niu S
Noack B
Nolan M
Noonan M https://orcid.org/0000-0003-4512-0535
North MA https://orcid.org/0000-0001-8794-4458
Nosrati H https://orcid.org/0000-0003-1509-9819
Novas F
Novosolov M
Nugnes F
Nürnberg D https://orcid.org/0000-0001-8644-3424
Obolski U https://orcid.org/0000-0001-7594-9745
O'Brien C
Ochsendorf J
Ogale A
Ogden R
O'Hara M
Ohe T https://orcid.org/0000-0002-2679-8191
Oikawa S
Oldham S
Oliveira AAM https://orcid.org/0000-0002-2826-5778
Oliveira-Filho F
Oliver P https://orcid.org/0000-0003-4291-257X
Olivieri AC
Olmedo D
Olson I
Omar MA
Ong YC
Opsenica I
Orben A https://orcid.org/0000-0002-2937-4183
Orgeret F https://orcid.org/0000-0002-1940-7797
Ormerod JT
Ortega JM
Ortí G
Ortiz J
Orzack S
Osth A
Ostojić L
Otani T
Owen D
Oyervides-Muñoz E
Page-Karjian A
Pagnotta MA
Pal U https://orcid.org/0000-0003-2110-4610
Pacchioni G http://orcid.org/0000-0002-4749-0751
Pallmann P
Palmeirim J
Palmer C https://orcid.org/0000-0002-1058-3428
Pan G
Pan S https://orcid.org/0000-0001-9080-5651
Panderi I
Pandit P https://orcid.org/0000-0001-7649-0649
Panigrahi P https://orcid.org/0000-0003-1365-5252
Papachristoforou A
Papageorgiou D
Papastamatiou Y https://orcid.org/0000-0002-6091-6841
Parameswaranpillai J
Park SS
Parks S https://orcid.org/0000-0001-6663-627X
Parveen S
Pasetto D https://orcid.org/0000-0001-6892-9826
Patel S
Paterson K https://orcid.org/0000-0002-8150-8628
Pauli F
Pearse D
Peddemors V
Pelella A
Peña J https://orcid.org/0000-0002-4137-1823
Peng H
Peng W
Penny A
Pepperberg I https://orcid.org/0000-0001-9486-7323
Perea M
Pereg M
Pereira D
Perelló J https://orcid.org/0000-0001-8533-6539
Perez-Garcia A
Perez-Huerta A
Perez-Saez J
Pernek M
Peron G https://orcid.org/0000-0002-6311-4377
Perumal S
Peterka R
Peterson D https://orcid.org/0000-0002-5673-4826
Petrulevičius JF
Pettinotti L
Photopoulou T https://orcid.org/0000-0001-9616-9940
Pianzola F https://orcid.org/0000-0001-6634-121X
Pickering J https://orcid.org/0000-0002-7242-9207
Piel A
Piella J
Piga G
Pincebourde S
Pinto L
Pires A
Pirotta E https://orcid.org/0000-0003-3541-3676
Plötner M
Pohjoismäki J https://orcid.org/0000-0002-1185-3610
Polak S
Polat NH https://orcid.org/0000-0002-2782-0581
Polet D https://orcid.org/0000-0002-8299-3434
Pollington T https://orcid.org/0000-0002-9688-5960
Pope E https://orcid.org/0000-0001-5781-5575
Porro L
Postolache D
Potticary A https://orcid.org/0000-0002-1157-5315
Pötzsche C
Pradhan S
Prado R
Prager K
Prague M
Prakash VCA
Prangle D
Preedy K
Preisig B
Price D https://orcid.org/0000-0003-0076-3123
Priede J
Prieler RJ
Prieto-Marquez A
Pritchard A
Promislow D https://orcid.org/0000-0001-7088-4495
Prosen H
Pugazhendhi A
Pugliese A
Pulkkinen K
Pullano G
Pungetmongkol P
Purnell M
Qader H https://orcid.org/0000-0001-8098-5994
Qian J
Qu Z
Quinn G
Rabi M
Racsmány M
Radchenko E
Ragab MAA
Rahbar N https://orcid.org/0000-0003-0159-0025
Rahman O
Rai V
Raihani N https://orcid.org/0000-0003-2339-9889
Rake B
Ramirez H
Ramirez M
Ramirez-Santiago G
Ramūnas Ž
Rapisarda A https://orcid.org/0000-0001-8290-8183
Rasalingam S
Rawlence N https://orcid.org/0000-0001-6968-6643
Raymundo L
Reber S https://orcid.org/0000-0001-8015-2538
Rebuli N https://orcid.org/0000-0003-2339-974X
Reddy A
Regolin L https://orcid.org/0000-0001-8960-0309
Reid B
Reimer J
Ren ZH
Resende BD
Reynolds Kirby A https://orcid.org/0000-0002-9678-8503
Reynolds J https://orcid.org/0000-0003-1536-1557
Reznikov N https://orcid.org/0000-0001-5293-4647
Riabinina O
Ribak G
Richards G
Richards H
Riordan P
Risse B
Ristig S
Roast M https://orcid.org/0000-0002-9454-7562
Robbins M
Robin N https://orcid.org/0000-0002-1756-3171
Rodríguez-Martínez J https://orcid.org/0000-0002-9543-2839
Roffet-Salque M
Rohrer J
Roleda M https://orcid.org/0000-0003-0568-9081
Romano P http://orcid.org/0000-0002-6819-2729
Rong L
Rong Z
Roohnia M https://orcid.org/0000-0002-7466-995X
Roozenbeek J https://orcid.org/0000-0002-8150-9305
Rosa-Garcia A https://orcid.org/0000-0003-2223-007X
Rosati A https://orcid.org/0000-0002-6906-7807
Roses M
Rosipal R
Ross C
Ross SD
Rössner G
Rout CS
Rovatsos M
Rowland M
Roy S
Royle S https://orcid.org/0000-0001-8927-6967
Rubin-Blum M
Rudnicki K
Rusconi E https://orcid.org/0000-0003-2700-205X
Russo N
Rychtar J https://orcid.org/0000-0001-6600-2939
Ryder OA
Rydin H https://orcid.org/0000-0002-7582-3998
Saar M https://orcid.org/0000-0002-2734-1871
Sabaté N
Sacco I
Sackur J
Saha B https://orcid.org/0000-0001-6711-0763
Sailer U https://orcid.org/0000-0002-9728-8738
Sainani K https://orcid.org/0000-0003-0614-303X
Saito A
Sajjadifar S
Sakiyama T
Salami E https://orcid.org/0000-0003-1748-6812
Saleh N
Samanta GK
Samuelson L
Sanchez N https://orcid.org/0000-0002-6467-1781
Sanchez-Amaro A https://orcid.org/0000-0003-4036-2455
Sandak A
Sanders SM
Santabarbara S
Santhanakrishnan A https://orcid.org/0000-0003-1800-8361
Santhi N
Sao H
Sapudom J
Saracco JF
Sarafoglou A
Sarkar S https://orcid.org/0000-0002-2037-0979
Sassi F
Sathe B https://orcid.org/0000-0001-8989-0967
Sato S
Sauermann J
Saxon J
Schaerf T
Scharf I https://orcid.org/0000-0002-8506-7161
Schedl M https://orcid.org/0000-0003-1706-3406
Scheiner S
Schellenberg G https://orcid.org/0000-0003-3681-6020
Schertzer E
Schiffbauer J
Schiller C
Schilling M
Schlaghecken F
Schneider CR
Schnetz L
Schöll E https://orcid.org/0000-0003-3372-207X
Schroeder R https://orcid.org/0000-0002-0613-3440
Schubnel A
Schuett W
Schulte P https://orcid.org/0000-0002-5237-8836
Schuster CS
Schwanz L
Schwen LO https://orcid.org/0000-0003-0195-9603
Schwieterman E
Scott R
Segalini A https://orcid.org/0000-0001-8667-0520
Selvaraj G
Semmens-Wheeler R
Semprebon G
Semwal VB
Senapati D
Seneca F
Sengupta D
Sengupta N
Sensuła B
Seto M https://orcid.org/0000-0003-4709-5206
Shaaban H
Shafiq Z
Shahamirian M
Shahrezaei V
Shankar K
Shannon G https://orcid.org/0000-0002-5039-4904
Sharbafi M https://orcid.org/0000-0001-5727-7527
Sharif MZ https://orcid.org/0000-0002-8980-8713
Sharma A
Sharot T
Sharp A https://orcid.org/0000-0001-5117-5335
Sharp V
Shawkey M https://orcid.org/0000-0002-5131-8209
Sheltzer J
Shepherd M
Sherbakov D
Sheridan I https://orcid.org/0000-0002-9149-7715
Sherry F https://orcid.org/0000-0003-4809-7254
Shi S http://orcid.org/0000-0001-8984-9809
Shi X
Shi Z https://orcid.org/0000-0003-2388-6695
Shikuku V
Shimabukuro YE
Shine B
Shipley O https://orcid.org/0000-0001-5163-3471
Shuang S
Siddheshwar P https://orcid.org/0000-0002-8932-2131
Siddiki SMAH
Siddique I
Sigaeva T
Sills J
Simon M
Simons A https://orcid.org/0000-0002-0198-465X
Singh A
Singh B
Siniscalchi M https://orcid.org/0000-0002-1592-6549
Skariyachan S
Skomal G
Sladky R
Smeijers D
Smith D
Smith JA https://orcid.org/0000-0003-2634-0268
Smith M https://orcid.org/0000-0002-8110-4367
Smith T
Snively E
Soares C
Sockman K https://orcid.org/0000-0002-3846-5386
Soda S
Sogani M
Solazzo C https://orcid.org/0000-0001-5092-0807
Sole'-Ribalta A
Solomon NG
Sontag A https://orcid.org/0000-0002-7088-0491
Sookias R https://orcid.org/0000-0002-5189-4011
Soto-Centeno JA https://orcid.org/0000-0002-1729-1153
Southall E https://orcid.org/0000-0002-7319-3080
Souza A
Soylak M
Spalding H
Sparagon W
Spasojevic P
Speakman C https://orcid.org/0000-0002-0023-518X
Speller C https://orcid.org/0000-0001-7128-9903
Spindler F
Spinelli JE
Sridhar V
Srinivasa Rao S https://orcid.org/0000-0002-1308-7067
Srivastava S
Srivastava VC
Srivathsa A https://orcid.org/0000-0003-2935-3857
St Clair M
Stack Whitney K
Stacy B
Stadie N
Stafford T
Steacy L
Stecher J
Steinbauer M
Stemler T
Stevenson J
Stickland R https://orcid.org/0000-0003-3398-4272
Stöhr S https://orcid.org/0000-0002-2586-7239
Story J https://orcid.org/0000-0002-1022-9381
Stovall A
Strauss E https://orcid.org/0000-0003-3413-1642
Strek T
Stringaris A
Strunk J
Struyve W
Sturla F https://orcid.org/0000-0001-7317-304X
Stuurman N
Su E
Suel E https://orcid.org/0000-0001-9246-3966
Sues H-D
Sugaya M
Suharyadi E
Sujith RI
Sumesh C
Sun H
Sun Y
Suresha B
Sutton G
Suzuki R
Swallow B https://orcid.org/0000-0002-0227-2160
Swaminathan S
Swarbrick HA
Swathy RS
Szolnoki A
Szreter S
Tabata J https://orcid.org/0000-0001-9095-2694
Taheri S
Tai XH
Taipale J https://orcid.org/0000-0003-4204-0951
Tait S
Tamè L
Tamietto M
Tang H
Tang J
Tang J
Tang S https://orcid.org/0000-0002-3324-746X
Tao X
Tasaki S
Tasoff J
Tay H
Taylor A
Tello-Aburto R
Tepole AB
Terashima I
Thewissen JGMH
Thomas CS
Thomas P https://orcid.org/0000-0003-4919-8452
Thompson C
Thompson HE
Tian GY
Tierney N
Tilburg WAP https://orcid.org/0000-0002-9724-0603
Timofeeva M
Tinios P
Tlili S
Toczyłowska-Mamińska R
Todd P
Togo K
Toledo N
Tomer V
Tönsing C
Tornabene F
Touloupakis E
Tran HN
Tranos E https://orcid.org/0000-0002-9620-6542
Tressoldi P https://orcid.org/0000-0002-6404-0058
Tricarico E
Tripathy SK
Trontelj J
Trostchansky A
Trotter J
Trueman C
Trujillo J https://orcid.org/0000-0003-4713-376X
Tryjanowski P https://orcid.org/0000-0002-8358-0797
Tsaousis A
Tschinkel W
Tsoi L
Tsuchiya N
Tudisco E
Turner N https://orcid.org/0000-0002-8708-0781
Tyler N
Tzavella L https://orcid.org/0000-0002-1463-9396
Ueda K
Ulhaq ZS https://orcid.org/0000-0002-2659-1940
Ulku HA https://orcid.org/0000-0003-4682-3902
Ullah S
Ummadisetti C
Uren H
Uyeno Y
Vadillo M https://orcid.org/0000-0001-8421-816X
Vaidya NK
Vaidya T https://orcid.org/0000-0002-2264-2595
Vajna B
Vallino J
Van den Bedem N
Van der Giessen E
Van Dongen N
Van Harreveld F
Van Mulukom V https://orcid.org/0000-0002-0549-7365
Van Strien M
Vannier J https://orcid.org/0000-0003-0998-1231
Vanpaemel W https://orcid.org/0000-0002-5855-3885
Vargas-Bernal R https://orcid.org/0000-0003-4865-4575
Varotto S
Vasquez V
Vega López MA
Velázquez MM
Veling H
Veller C
Vences M
Venkatraman RK https://orcid.org/0000-0003-0636-5310
Verbruggen S
Veríssimo A https://orcid.org/0000-0003-3396-9822
Verkerk A https://orcid.org/0000-0002-3351-8362
Vernes S https://orcid.org/0000-0003-0305-4584
Versace E
Vignoles V
Villegas NM
Vinauger C
Vincent B
Virgilio M
Vladimiro C
Vonk J
Waclaw B https://orcid.org/0000-0001-5355-7994
Wada H
Wagner G https://orcid.org/0000-0003-1086-2301
Wahba MEK https://orcid.org/0000-0002-1352-0297
Walke DP
Walker D
Walker J
Wall C
Walsh MK
Waltman L
Wanapat M
Wang H-H
Wang J https://orcid.org/0000-0002-1371-6425
Wang K
Wang P
Wang VY https://orcid.org/0000-0003-0895-3132
Wang W
Wang X
Wang X
Wang X
Wang X
Wang Y
Wang Y https://orcid.org/0000-0002-1336-7464
Wang Y
Wang Z
Wang Z
Ward TA
Watkins G
Watling L
Watson S
Watts D https://orcid.org/0000-0001-6603-2230
Wechsung F
Wedekind C https://orcid.org/0000-0001-6143-4716
Wegener S
Wegner KM https://orcid.org/0000-0002-2410-8898
Wei J
Wei Z
Weinstein N https://orcid.org/0000-0003-2200-6617
Welch K
Wen H https://orcid.org/0000-0002-2045-729X
Weslawski J
Wessells R
West M
Wheate NJ
White D https://orcid.org/0000-0002-6366-2699
White PS
Whiteley N
Whiteman J
Whitlock M
Whitney M https://orcid.org/0000-0002-6671-5127
Whittaker D https://orcid.org/0000-0002-3491-5642
Whittles L
Wicherts J
Wieczorek PP
Wigneshweraraj R
Wilhelm O
Wilkinson A
Williams B
Wilts B https://orcid.org/0000-0002-2727-7128
Wittmann J
Wolf C
Wolf W https://orcid.org/0000-0003-4379-1343
Wolff G
Wolfram U
Wolsan M https://orcid.org/0000-0002-2083-7743
Wong B
Wood J
Woodward A
Woodward J
Wu A https://orcid.org/0000-0002-1801-5716
Wu R-L
Wu S
Wu Y
Wu Y
Wuttke A https://orcid.org/0000-0002-9579-5357
Wypych F
Xia C-Y
Xie L-H
Xie S
Xu F
Xu Y-J
Yamamoto S
Yamauchi Y
Yang A https://orcid.org/0000-0001-5974-247X
Yang B
Yang C https://orcid.org/0000-0001-9184-5030
Yang Y https://orcid.org/0000-0003-0576-6032
Yao C
Yap KN
Yasar S
Yau CY
Ydenberg R
Yeomans M
Yin X
Yokozawa T
Young B
Young J https://orcid.org/0000-0002-8671-990X
Young M
Yousefi A https://orcid.org/0000-0002-9096-3147
Yu L
Yu S
Yuan Y
Yuan Z
Yue C
Yun H
Yusuf A https://orcid.org/0000-0002-8625-6490
Zakaria M https://orcid.org/0000-0003-2456-6415
Zanon M
Zare-Dorabei R
Zauscher S
Zeng H https://orcid.org/0000-0002-5728-7896
Zhang F
Zhang G
Zhang L
Zhang Q https://orcid.org/0000-0002-7461-9880
Zhang S https://orcid.org/0000-0003-0170-3835
Zhang W
Zhang X
Zhang Y
Zhang Y
Zhao C-S
Zhao Q
Zhao Y-G
Zhou M
Zhou S
Zhou W-H
Zhou X
Zhou Y
Zhu J
Zhu KY
Zino L https://orcid.org/0000-0002-0946-6523
Zivanovic S
Zwaka H https://orcid.org/0000-0002-4469-5719

